# The Envelope Nuisance

**Published:** 1871-05

**Authors:** 


### THE ENVELOPE NUISANCE.

An envelope containing a letter written by one who has the small-pox may communicate the dreadful disease.

A man's heart may sink within him, if, while struggling to get a foothold in the world, he calls at the post-office for a letter, and is handed one with a lawyer's address printed in one corner. Respectable lawyers do not need to publish their vocation in order to get business.

But of all the inconsiderate, ruthless, foolish, hypocritical, dangerous, and cruel devices, none exceeds that of sending letters with a black border around them, — "mourning envelopes." Scarcely a reader but will confess to an undefinable uneasiness, if not an actual shock, to be kept up until the letter is opened, and the name at the bottom deciphered. The very sending of a mourning letter to any one is a barbarity, a brutality, a hypocrisy, and a lie. True grief detests display; the deeper the sorrow, the more intense is the desire to shun observation, to shrink within itself, to shut out all creation. The world over, the men who mourn the loudest for their lost wives, are in the greatest haste to get married again. None is happier than the extravagant heir, whose father has just died, and left him untold thousands; his envelopes will have black borders a foot broad for the next ten years.

A poor woman, whose husband was at the "front" during the war, had been threatened by her landlord to be turned out of doors next day, unless the rent was paid; she had been anxiously looking for a remittance from her brave husband, but it did not come; in the early morning, before she had left her bed, her babe at her side, there was a knock at the door. "There's the landlord," she exclaimed, and the next instant was a corpse. An emotion kills. This was an emotion of apprehension, and countless numbers of these, with pangs only a little less fatal, are uselessly occasioned every day in many a trembling heart, in many a nervous temperament, by this same sending of "mourning envelopes." Reader, if you have a spark of true humanity, you will never send another.

